# Psychological characteristics of pedagogical activity of scientists

**DOI:** 10.1192/j.eurpsy.2022.1947

**Published:** 2022-09-01

**Authors:** A. Gasimov, A. Konovalova, K. Maslova

**Affiliations:** 1 Lomonosov Moscow State University, Faculty Of Psychology, Moscow, Russian Federation; 2 I.M. Sechenov First Moscow State Medical University, Department Of Pedagogy And Medical Psychology, Moscow, Russian Federation

**Keywords:** modern education, pedagogical competences, success of the teacher

## Abstract

**Introduction:**

Appeal to pedagogical abilities, important for the success of the teacher, allows us to highlight some actual requirements for the scientist.

**Objectives:**

The research is aimed at studying various competencies as a factor of success of the teacher.

**Methods:**

The method of work is a bibliographic analysis.

**Results:**

Firstly, they are communicative abilities, by which are meant the ability to communicate, the ability to find an approach to students, to build trustful dialogue.

Secondly, they are didactic abilities that make it possible to intelligently present the knowledge, stimulate interest in the subject, stimulate students ’cognitive activity, the ability to organize students’ independent work, and form their need for independent knowledge acquisition.

Thirdly, the academic pedagogical abilities of scientists, that is, the abilities for the corresponding field of science, the knowledge of the subject taught, not only in the volume of the training course, but much wider and deeper are the aspect of the necessary pedagogical competencies of scientists.

Fourthly, pedagogical abilities are related to the research orientation of the teacher, with his need and ability to conduct his own research work.

Fifth, among the pedagogical abilities, the pedagogical imagination is distinguished, presupposing the ability to project and predict the development of the student’s actions.

**Conclusions:**

It can be concluded that modern education and society as a whole formed a new request to science and scientists, consisting in the development of their respective pedagogical competences.

**Disclosure:**

No significant relationships.

